# Upregulation of Mir-21 Levels in the Vitreous Humor Is Associated with Development of Proliferative Vitreoretinal Disease

**DOI:** 10.1371/journal.pone.0158043

**Published:** 2016-06-28

**Authors:** Ayumi Usui-Ouchi, Yasuo Ouchi, Masatoshi Kiyokawa, Toshiro Sakuma, Rei Ito, Nobuyuki Ebihara

**Affiliations:** 1 Department of Ophthalmology, Juntendo University Urayasu Hospital, Urayasu, Chiba, Japan; 2 Division of Innate Regulation, International Research, and Development Center for Mucosal Vaccine, The Institute of Medical Science, The University of Tokyo, Tokyo, Japan; University of Massachusetts Medical, UNITED STATES

## Abstract

MicroRNAs (miRNAs) are small noncoding RNAs that regulate gene expression by post-transcriptional inhibition of mRNA translation. Dysregulation of miRNAs, including circulating miRNAs, has been reported to play an important role in the development of various diseases, including fibrotic diseases. Aberrant expression of miRNAs in the vitreous humor of vitreoretinal diseased eyes has been reported. However, the expression pattern of miRNAs present in the vitreous humor of proliferative vitreoretinal disease (PVD) patients, including proliferative diabetic retinopathy (PDR), and proliferative vitreoretinopathy (PVR), remains unknown. To investigate the factors important for the development of PVD, we characterized the miRNAs present in the vitreous humor of PVD patients and analyzed the expression profiles of 377 miRNAs using quantitative polymerase chain reaction-based miRNA arrays. The expression of a specific subset of miRNAs, previously reported to be associated with the development of angiogenesis and fibrosis, was significantly altered in the vitreous of PVD patients. Among these miRNAs, we identified miR-21 as a candidate fibrotic miRNA with an important role in the pathogenesis of PVD. Increased miR-21 levels in the vitreous were associated with retinal fibrosis, including PVR and PDR. Because epithelial-mesenchymal transition (EMT) of retinal pigment epithelial cells (RPECs) plays a critical role in retinal fibrosis, the expression of miR-21 in human RPECs was determined. Its expression in RPECs was induced by transforming growth factor-β, a key growth factor involved in fibrogenesis, and was enhanced by high glucose culture conditions, suggesting that miR-21 expression positively correlates with disease progression. Gain- and loss-of-function studies revealed that miR-21 promoted cell proliferation and migration of ARPE-19 cells without affecting EMT-related gene expression. Together, our studies have identified miR-21 as a potential disease-modifying miRNA in the vitreous humor that is involved in the development of retinal fibrosis and may be a novel marker of PVD.

## Introduction

Proliferative vitreoretinal diseases (PVDs), including proliferative diabetic retinopathy (PDR) and proliferative vitreoretinopathy (PVR), are leading causes of blindness due to tractional retinal detachment resulting from a fibroproliferative response caused by increases of various biologically active growth factors in the eye [1]. Surgical approaches for the treatment of these disorders have significantly improved in the recent past. However, the occurrences of fibrotic responses, such as cicatricial contraction of proliferative membranes, limit the therapeutic success.

Although a number of studies have emphasized the molecular basis of retinal fibroproliferative disease [1–4], information is still insufficient to develop an effective treatment, and optimal management for retinal fibroproliferative disease has not been established. The microRNAs (miRNAs) are a specific class of noncoding RNAs that are defined as small (approximately 20 nucleotides in length) RNAs that are processed from a much larger primary transcript. Once processed into their mature forms, miRNAs generally bind to complementary sequences at the 3′ untranslated region of specific genes. The miRNAs mediate silencing of their bound targets via mRNA destabilization and/or protein translation inhibition, and play critical roles in various biological processes, such as proliferation, differentiation, apoptosis, immune function, and angiogenesis [5]. Recently, miRNAs have been found in various kinds of body fluids, such as serum, plasma, saliva, tears, urine, and breast milk [6].

The importance of miRNAs in the extracellular space has been confirmed by a number of studies reporting specific and regulated export from the cell of exosome-mediated miRNAs with thermal and acid stability, and their uptake and functional consequences in recipient cells [7–10]. In addition to providing a strategy for the delivery of drugs or RNA therapeutic agents, exosomal components can serve as biomarkers that aid in diagnosis, to help determine treatment options and prognoses. Furthermore, recent studies have shown that miRNAs are also present in the vitreous and aqueous humors of the eye, and some miRNAs have been reported to be closely associated with the development of vitreoretinal disease, such as vitreoretinal lymphoma and diabetic retinopathy [11–14]. However, it remains unclear whether disease-specific miRNAs exist in the vitreous humor of PVDs, and/or play a role in the development of the disorder.

In this study, we report a comprehensive characterization of miRNA expression changes in the vitreous humor of PVD patients. The results show that the expression of a specific subset of miRNAs, previously reported to be associated with the development of angiogenesis and fibrosis, is significantly altered in the vitreous humor of eyes of PVD patients. Among these miRNAs, we identified microRNA-21 (miR-21) as a candidate fibrotic miRNA present in the vitreous humor with an important role in the pathogenesis of PVD, involving control of proliferation and migration of retinal pigment epithelial cells (RPECs).

## Materials and Methods

### Patients and Samples

All procedures involving patients in this study adhered to the Declaration of Helsinki. Vitreous humor samples were collected from three patients with macular hole (MH) designated as the control group (two males and one female, 54–69 years of age) and three patients with PDR having tractional detachment with active fibrovascular membrane (PDR with PVR) designated as the PVD group (two males and one female, 46–64 years of age) for miRNA array studies. The vitreous of seven patients with MH, 10 patients with PDR with PVR, five patients with rhegmatogenous retinal detachment (RRD), and five patients with PVR were also collected to validate the miRNA assays. All patients underwent vitrectomy at the Juntendo University Urayasu Hospital between May 2013 and September 2014. Patients with systemic diseases such as autoimmune diseases and cancer, and with histories of ocular diseases and/or ocular surgeries were excluded from both groups.

All surgeries were performed by an experienced vitreoretinal surgeon (M.K.). A 25-gauge trocar was inserted 4 mm posterior to the corneal limbus using a transconjunctival one-step incision before the start of surgery. Vitreous samples were collected before the infusion, and immediately centrifuged at 200 × *g* for 10 min to eliminate any cell debris. All samples were immediately frozen and stored at −80°C until the assays were performed.

### Ethics

The study was approved by the Juntendo University Urayasu Hospital Research Ethics Committee (2011–045). Eligible participants were informed about the purpose and experimental procedure of the study, and signed a copy of the Juntendo University Urayasu Hospital Research Ethics Committee approved consent form prior to participation.

### RNA Extractions

The frozen vitreous humor samples were thawed on ice, and the supernatant was collected by centrifugation at 3,000 × *g* for 5 min at 4°C. Total RNA was extracted from the resultant supernatants using the miRNeasy™ Serum/Plasma Kit according to the manufacturer’s protocol (Qiagen, Hilden, Germany). Briefly, 200 μL aliquots of the vitreous humor samples were mixed with 1,000 μL of QIAzol™ Lysis Reagent (Qiagen) and incubated for 5 min. Then, 200 μL of chloroform was added, vigorously mixed, and incubated for 2 min. Samples were centrifuged at 12,000 × *g* for 15 min at 4°C, and the upper aqueous phase was collected and mixed with 900 μL of 100% ethanol. The contents were mixed thoroughly, and 700 μL of the sample was transferred to a Qiagen RNeasy^®^ MinElute Spin Column and purified according to the manufacturer’s protocol. The final total RNA elution volume was 14 μL, and the total RNA was stored at -80°C. For RNA purification from cell cultures, total RNA was extracted by using the RNeasy™ Mini Kit according to the manufacturer’s protocol.

### TaqMan Array MiRNA Cards

The miRNA expression profiling was performed on six samples (three samples from MH patients and three samples from PDR with PVR patients) using the TaqMan^®^ Human MicroRNA Array A Cards (Applied Biosystems Life Technologies, Carlsbad, CA, USA) according to the manufacturer’s protocol. Briefly, 3 μL of total RNA from each sample was reverse transcribed using the TaqMan^®^ miRNA Reverse Transcription Kit (Applied Biosystems) and the stem-loop Megaplex^™^ Primer Pool Sets (set A). A total of 7.5 μL of reaction mixture was immediately incubated under the following conditions: 40 cycles at 16°C for 2 min, 42°C for 1 min, and 50°C for 1 s. Then, 2.5 μL of the resultant Megaplex^™^ RT products were mixed with 2.5 μL of Megaplex^™^ PreAmp Primers (pool A) and 12.5 μL of TaqMan^®^ PreAmp Master Mix. A total of 25 μL of the reaction mixture was incubated using the following program: 95°C for 10 min, 55°C for 2 min, and 75°C for 2 min followed by 12 cycles at 95°C for 15 s and 60°C for 4 min. The preamplified cDNA was diluted with 0.1× TE (10 mM Tris, pH 8.0, 1 mM EDTA) to 100 μL and used for real-time polymerase chain reactions (PCRs).

The expression profiles of miRNAs were acquired using TaqMan^®^ Low Density Arrays according to the manufacturer’s instructions. Raw cycle threshold (C_t_) values were calculated using SDS software, version 2.3 (Applied Biosystems) and applied automatic baseline settings with an assigned minimum threshold of 0.2. Because a C_t_ value of 35 represents single template detection, samples with C_t_ values < 35 were considered as positive. For hierarchical clustering, ΔC_t_ values were log_2_ transformed and median-centered, and then cluster analyses of differentially expressed miRNAs were performed using Manhattan correlation as a quantification of similarities. All data analyses were performed using Mev software (MultiExperiment Viewer; TM4, Boston, MA, USA). The experimental data in this manuscript are freely available in the NCBI gene expression omnibus (GEO) repository with the accession number GSE74559.

### Quantitative Real-Time PCR

For quantitation of individual miRNAs, cDNA was synthesized from total RNA using miRNA-specific primers according to the manufacturer’s protocol for the TaqMan^®^ MicroRNA assay (Applied Biosystems). Briefly, reverse transcriptase reactions were performed in 15 μL containing 5 μL of purified total RNA, 50 nM stem-loop RT primers, 1× RT buffer, 0.25 mM each of dNTPs, 3.33 U/μL Multiscribe™ Reverse Transcriptase, and 0.25 U/μL of RNase inhibitor. The reverse transcription reaction mixtures were incubated for 30 min at 16°C, 30 min at 42°C, 5 min at 85°C, and then cooled. RT products were further diluted four times with RNase-free water. Real-time PCR was performed using TaqMan^®^ Universal PCR Master Mix. A 20-μL PCR reaction included 1 μL of diluted RT product, 1× of the corresponding miRNA assay primers, and 1× TaqMan^®^ Universal PCR Master Mix (Applied Biosystems). Real-time PCR reactions were performed using the Applied Biosystems 7900HT (Applied Biosystems) with the following conditions: 95°C for 10 min, followed by 50 cycles of 95°C for 15 s, and 60°C for 1 min. All reactions were run in duplicate. Real-time PCR was performed using the Applied Biosystems 7900HT (Applied Biosystems). Data were analyzed with SDS software, version 2.3 (Applied Biosystems), to determine C_t_ by the second derivative max method. Relative quantities of miRNAs were calculated using the ΔΔ*C*_t_ method with reference U6 snRNA (average C_*t*_ values of all four independent U6 snRNA probes) as internal controls. The ΔC_t_ (C_*t*_ target miRNA—C_*t*_ U6 snRNA average) relative expression = 2^−ΔΔCt^, where ΔΔCt = (ΔCt of the sample)—(ΔCt of the calibrator).

For mRNA quantification, quantitative PCR was performed using standard protocols with an Applied Biosystems 7900HT thermocycler. Briefly, cDNA was synthesized from total RNA using PrimeScript RT Master Mix (Takara, Shiga, Japan). Quantitative PCR was then performed using a SYBR Premix Ex Taq II (Tli RNaseH Plus; Takara). GAPDH was used for the normalization of mRNA levels, and relative quantification was performed using the comparative ΔΔ*C*_t_ method. The primer sequences used for the PCR are shown from 5′ to 3′ as follows:

Fibronectin, (forward, GGAGAATTCAAGTG TGACCCTCA; reverse, TGCCACTGTTCTCCTACGTGG); αSMA, (forward, CCGACCGAATGCAGAAGGA; reverse, ACAGAGTATTTGCGCTCCGGA); N-cadherin, (forward, CGAATGGATGAAAGACCCATCC; reverse, GGAGCCAC TGCCTTCATAGTCAA); and GAPDH, (forward, GCAAATTCCATGGCACCGT; reverse, TCGCCCCACTTGATTTTGG).

### Cell Culture and Transfection

The immortalized human RPE cell line, ARPE-19, was purchased from the American Type Culture Collection (Manassas, VA, USA). The cells were cultured in DMEM/F12 medium supplemented with 10% fetal bovine serum, 50 U/mL penicillin, and 50 μg/mL streptomycin at 37°C in an atmosphere of 5% CO_2_ [15].

The effect of transforming growth factor-β (TGF-β) on ARPE-19 cells was examined by adding 10 ng/mL recombinant human TGF-β2 (R&D Systems, Minneapolis, MN, USA) to growth-arrested cell monolayers at 70% confluency. For high- or low-glucose treatments, ARPE-19 cells were cultured in DMEM/Ham’s F-12 medium supplemented with 30 mM glucose (high) or with 5 mM glucose (low) and 30 mM mannitol.

For transfection with the mirVana miR-21 mimic or the miR-21 inhibitor (Ambion, Foster City, CA, USA), the siPORT™ NeoFX™ transfection reagent (Applied Biosystems) was used according to the manufacturer’s instructions. Briefly, ARPE-19 cells were seeded to 70% confluency and then transfected with 10 μM of mirVana miR-21 mimic or miR-21 oligonucleotide inhibitors.

### Enhanced Green Fluorescent Protein (EGFP) Reporter Gene Based miRNA-Target Interaction Assay

CMV-EGFP-3'-UTR miR-21-target sequence reporter plasmid was created by cloning the miR-21 binding sequence into the XhoI/NotI site located at the 3'-UTR of pcDNA3.1-EGFP. The oligonucleotide sequences used for the construction are shown from 5' to 3' as follows: miR-21 target forward, GGCCGCTCAACATCAGTATGATAAGCTAT; miR-21 target reverse, CTAGATAGCTTATCAGACTGATGTTGAGC.

ARPE-19 cells were seeded onto 12-well plates at 2 × 10^6^ cells/plate. After 12 h, the cells were co-transfected with a combination of 1 μg of pcDNA-EGFP or pcDNA-EGFP-miR21-target and 10 μM of mirvana miR-21 mimic, mirvana miR-21 inhibitor, or a negative control miRNA. After 24 h, GFP expression was examined under a fluorescence microscope (BZ-9000; Keyence, Osaka, Japan) and fluorescent images (exposure time 1/15 s) were taken in at least five different places.

### Cell Migration Assay

ARPE-19 cells were seeded onto 12-well plates at 2 × 10^6^ cells/plate. After 12 h, the cells were transfected with 10 μM of mirvana miR-21 mimic, mirvana miR-21 inhibitor, or negative control miRNA using the siPORT™ NeoFX™ transfection reagent (Applied Biosystems). Once the cultures reached confluency, the cell monolayers were wounded using a 200 uL yellow pipette tip and cultured with or without 10 ng/mL recombinant human TGF-β2 (R&D Systems). Nine wounds were made for each sample, and migration distance was photographed (three points for each wound) and measured at zero time and after 12 h.

### Cell Proliferation Assay

Cell proliferation was assessed using the CCK-8 cell counting kit (Dojindo, Rockville, MD, USA) according to the manufacturer’s protocol. Briefly, miR-21 mimic or miR-21 inhibitor oligonucleotide-transfected ARPE-19 cells were seeded onto a 96-well plate at a concentration of 1 × 10^4^ cells/well. The CCK-8 reagent was added, and the absorbance was measured at 450 nm using a microplate reader at three time points, 24 h, 48 h, and 72 h posttransfection.

### Immunocytochemical Analysis

ARPE-19 cells were cultured on 4-well multi-chamber slides (EMD Millipore, Billerica, MA, USA) in serum-free medium for 12 h, then treated with 10 ng/mL recombinant TGF-β2 for 48 h. The cells were then fixed in 4% paraformaldehyde and incubated with primary antibodies. The primary antibodies used were as follows: anti-ZO-1(Invitrogen, Waltham, MA, USA) and anti-aSMA (Abcam, Cambridge, UK). Primary antibodies were visualized using appropriate secondary antibodies conjugated with Alexa Fluor 488 or 594 (Invitrogen). Nuclei were counterstained with 4',6-diamidino-2-phenylindole (DAPI). Fluorescence and differential interference contrast images were observed using a fluorescence microscope (BZ-9000; Keyence, Osaka, Japan).

### Statistical Analyses

The unpaired *t*-test (*p*-value < 0.05) was applied to statistically evaluate the expression differences between the MH and PVD groups, from the results of the TaqMan^®^ miRNA arrays. The Mann-Whitney U test and one-way analysis of variance with Turkey’s multiple comparison tests were used to statistically evaluate the expression differences between two groups and multiple groups, respectively, for the results of the single TaqMan^®^ miRNA assays.

## Results

### Identification of MiRNAs Differentially Expressed in the Vitreous Humor of PVD Patients Using Quantitative PCR Arrays

**Fig 1:**
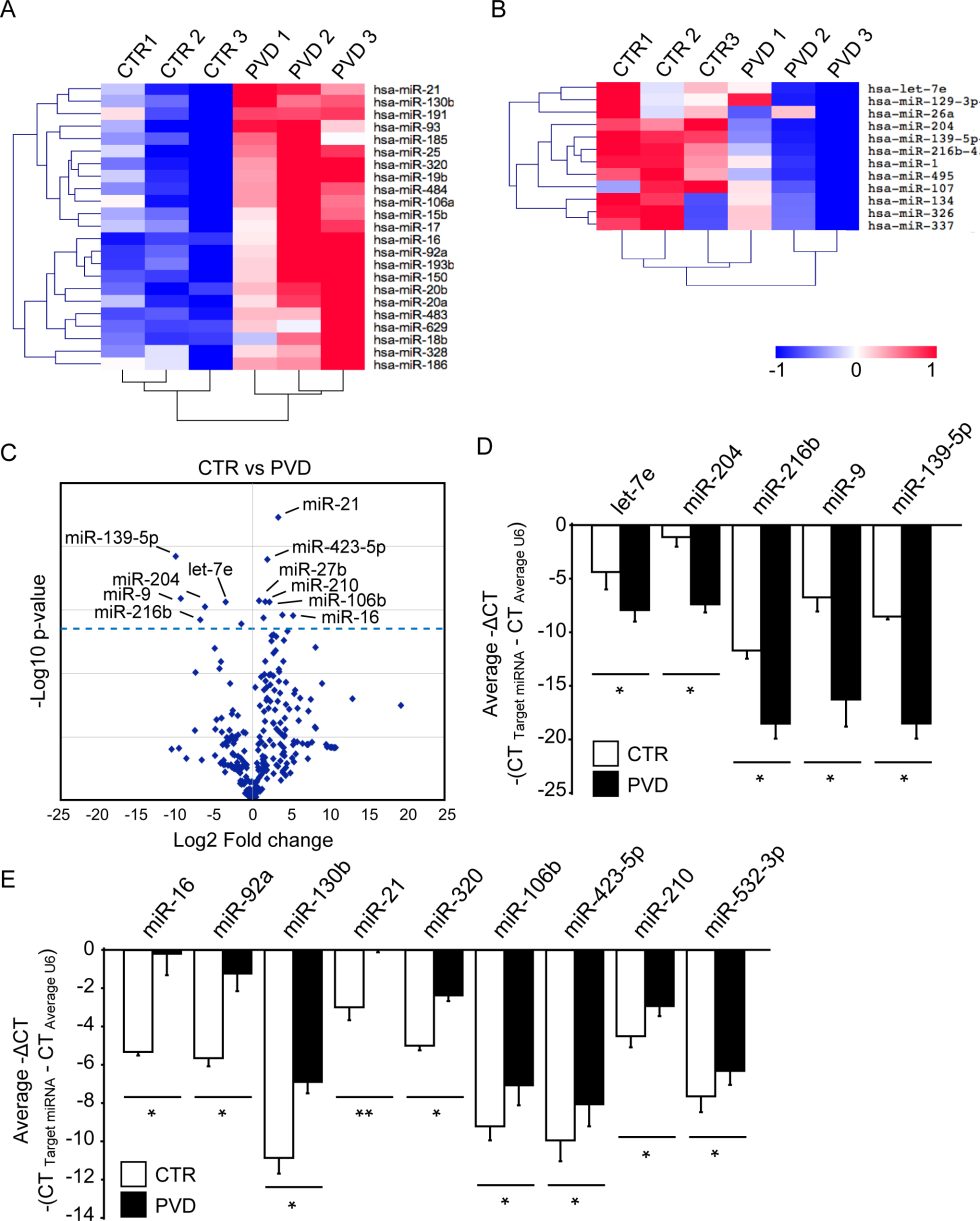
Comparison of expression levels of miRNAs in the PVD and MH samples. (A, B) Clustering analyses of the expression patterns of microRNAs (miRNAs) in the vitreous of PVD (n = 3) and control MH (n = 3) samples. Red indicates high expression and blue indicates low expression. (C) Volcano plot of real-time polymerase chain reaction (PCR)-based miRNA array data from the vitreous humor of PVD and control MH samples. Profiling for 377 miRNAs was performed on the vitreous humor of PVD (n = 3) and control MH (n = 3) samples. The y-axis depicts the negative log_10_ of *p*-values of the *t*-tests (the horizontal line corresponds to a *p*-value of 0.05; a higher value indicates greater significance), and the x-axis depicts the difference in expression between the two experimental groups as log_2_ fold changes. (D, E) Expression levels of miRNAs detected by real-time PCR-based miRNA arrays that were significantly different (*p* < 0.05) between PVD (n = 3) as compared with control MH (n = 3) samples. The average of ΔCt values of control MH samples (white bars) and proliferative vitreoretinal disease (PVD) samples (black bars) were calculated with standard deviations. To reverse the inverse relationship between ΔCt values and miRNA expression levels, ΔCt values were transferred into negative ΔCt values. The differences of ΔCt values between PVD and control MH samples are shown by relative expression levels of downregulated miRNAs (D) and upregulated miRNAs (E) in PVD. CTR; control, PVD; proliferative vitreoretinal disease; MH, macular hole. ** *p* < 0.01; *p < 0.05.

To obtain comprehensive miRNA expression profiles from the vitreous humors of PVD patients, we examined the expression profiles of 377 miRNAs from the vitreous humor of six patients affected by MH as the control group (three patients) and PDR with active fibrovascular membrane as the PVD group (three patients) using TaqMan^®^ Low Density Array technology. For the normalization of miRNA expression levels, we used average C_*t*_ values of all four independent U6 snRNA probes as internal controls. The average C_*t*_ values of U6 snRNA in MH and PVD samples were 21.4 ± 0.44 and 21.5 ± 1.36, respectively, which showed no significant difference. There was substantial expression of 232 miRNAs in the vitreous humor samples from MH patients, and 221 miRNAs in the vitreous humor samples of PVD patients. Approximately 46% of the miRNAs in the vitreous humor samples of the PVD group showed changes of more than 3-fold in expression compared with the control group. Hierarchical clustering of the fold-changes between the control and PVD groups showed distinct clusters and showed that 23 miRNAs were consistently upregulated in the PVD group (Fig 1A, S1 Fig), whereas 12 miRNAs were consistently downregulated (Fig 1B, S1 Fig).

To identify statistically significant differences in the expressions of miRNAs between the PVD and control groups, we performed a volcano plot filtering against these miRNA expression profiles. A change in miRNA expression was considered statistically significant if the fold change was > 2.0 and the *p*-value was < 0.05. As a result, we identified the expression levels of five miRNAs, including let-7e, miR-204, miR-216b, miR-9, and miR-139-5p as significantly downregulated in the PVD group, as shown by lower -ΔCT values (Fig 1C and 1D). The expression levels of nine miRNAs, such as miR-16, miR-92a, miR-130b, miR-21, miR-320, and miR-106b, were significantly upregulated in the PDV group (Fig 1C and 1E).

To investigate whether the miRNAs in PVD samples were regulating the target gene expressions in retinal fibroproliferative disorders, we analyzed the gene expression datasets in inactive fibrovascular membrane (FVM) and active FVM of PDR samples from the GEO (NCBI Geo; GSE60436). The regulatory relationships between the identified miRNAs and the differentially expressed genes in active FVM were analyzed using *in silico* functional analyses with DIANA-mirExTra. These data showed that the expressions of the identified miRNAs were negatively correlated with their predicted mRNA targets (S1 and S2 Tables), suggesting that these miRNAs were actively regulating mRNA expression in PVD patients.

### Altered Expression of MiR-21 Is Associated with the Pathogenesis of Retinal Fibroproliferation

**Fig 2:**
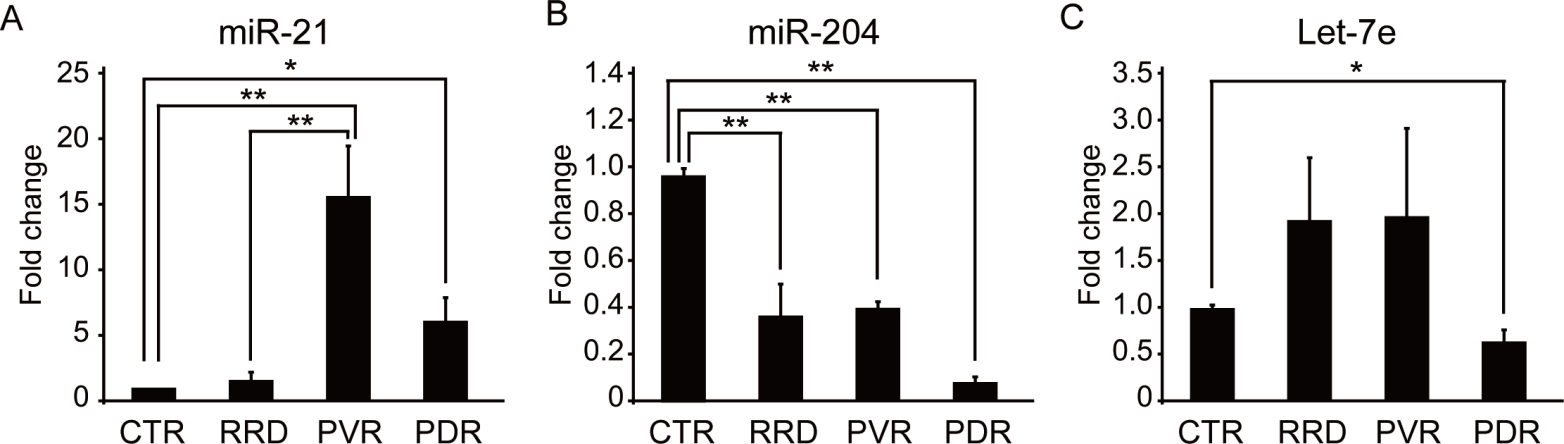
Comparisons of expression levels of miR-21, miR-204, and let-7e in the vitreous humor of MH, RRD, PVR, and PDR samples. (A, B, and C) Results of quantitative PCR (qPCR) analyses of miR-21, miR-204, and let-7e levels from the vitreous humor of MH (n = 7), RRD (n = 5), PVR (n = 5), and PDR (n = 10) samples. Fold changes in expression levels of miR-21(A), miR-204 (B), and let-7e (C) in RRD, PVR, and PDR samples were determined relative to the control MH sample. CTR, control; RRD, rhegmatogenous retinal detachment; MH, macular hole; PVR, proliferative vitreoretinopathy; PDR, proliferative diabetic retinopathy. **p < 0.01; *p < 0.05.

To identify the miRNAs associated with the pathogenesis of retinal fibroproliferation, we chose to focus on three miRNAs: miR-21 [16–25], miR-204 [26, 27], and let-7e [28, 29]. These miRNAs have previously been reported to play important roles in the regulation of tissue fibrosis, including idiopathic pulmonary fibrosis [20, 24], cardiac fibrosis [18], systemic sclerosis [22], interstitial fibrosis and tubular atrophy [26], and hepatic cirrhosis [17]. Detailed expression analyses of these three miRNAs in vitreous humor samples of MH patients as controls (n = 7), and in RRD patients (n = 5), PVR patients (n = 5), and PDR patients with active fibrovascular membranes (n = 10) were examined by TaqMan^®^ real-time PCR. The expression level of miR-21 in the vitreous humor of PDR patients was significantly higher than that of the control group (Fig 2A). However, the expression levels of miR-204 and let-7e in the vitreous humor of PDR patients were significantly lower than in the control group (Fig 2B and 2C).

When we examined the expression levels of miR-21, miR-204, and let-7e in the vitreous humor of primary fresh RRD samples without fibroproliferation, we found that the expression level of miR-204 in the vitreous humor of RRD eyes was significantly lower than that of the controls, but there were no obvious differences between the RRD and PVR eyes (Fig 2B).

The expression level of miR-21 in the vitreous humor of PVR eyes was significantly higher than MH eyes, and also higher than RRD eyes (Fig 2B). The expression level of let-7e in the vitreous of PVR eyes was not significantly changed compared with MH and RRD eyes (Fig 2B). These results suggested that the altered expression levels of miR-21 were associated with the pathogenesis of retinal fibroproliferation.

### Expression of MiR-21 and MiR-204 Is Regulated by TGF-β in Retinal Pigment Epithelial Cells

The pathogenesis of PVD patients, such as PVR and PDR with active fibrovascular membranes, begins with the enhanced proliferation and EMT of RPECs located in the vitreous cavity and subretinal space, and with the excess production of the fibrillary extracellular matrix on the retinal surface occurring in PDR [30, 31]. During this process, it has been reported that TGF-β-induced EMT of RPECs plays an important role in disease progression [2].

**Fig 3:**
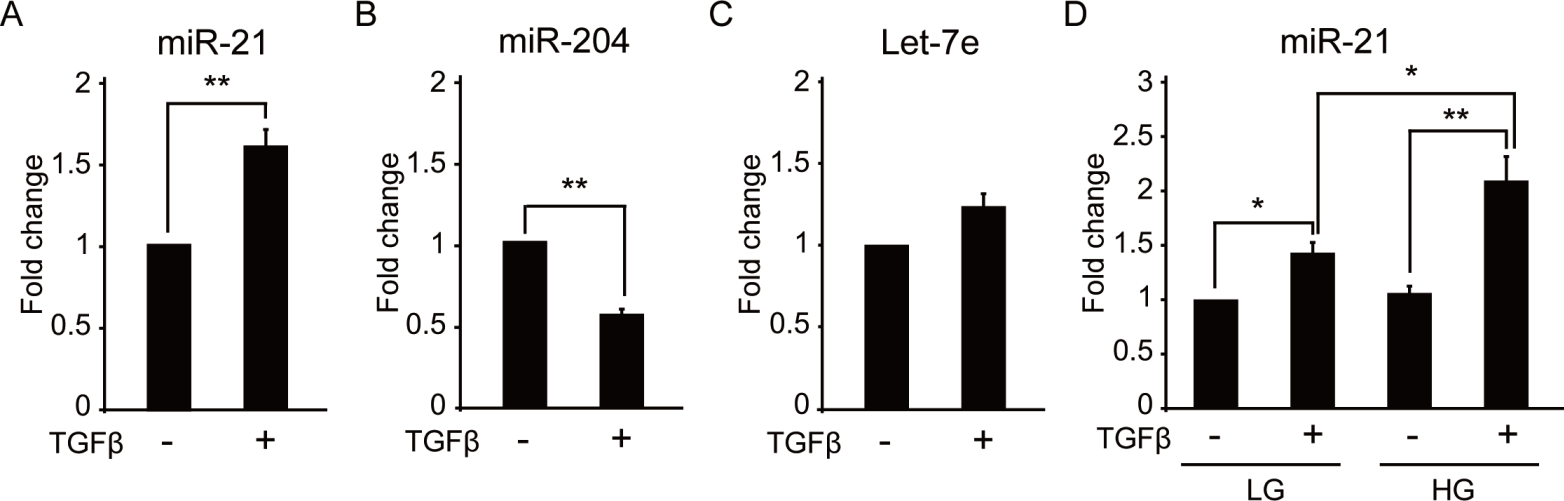
Expression of miR-21 is induced by signaling and high glucose conditions in RPECs. (A, B, and C) Results of quantitative (q) PCR analyses of miR-21, miR-204, and let-7e levels in TGF-β-stimulated ARPE-19 cells. Fold changes in expression levels of miR-21 (A), miR-204 (B), and let-7e (C) in TGF-β-stimulated ARPE-19 cells were determined relative to the unstimulated control cells. (D) Results of qPCR analyses of TGF-β-induced expression of miR-21 in ARPE-19 cells under low or high glucose conditions. Fold changes in expression levels of miR-21 in TGF-β-stimulated ARPE-19 cells under low or high glucose culture conditions were determined relative to the unstimulated control cells. RPECs, retinal pigment epithelial cells; TGF-β, transforming growth factor-β; LG = low glucose; HG = high glucose. **p < 0.01; *p < 0.05.

We therefore examined whether the TGF-β-induced EMT influenced the expression levels of miR-21 and miR-204 in RPECs. A human retinal pigment epithelial cell line (ARPE-19) was used as a model system and was stimulated with 10 ng/mL of human recombinant TGF-β2. Induction of EMT was examined by cell morphological and immunohistochemical analyses. A drastic change in cell morphology was apparent after 48 h of culture with TGF-β2, and immunohistochemical analyses revealed that the expressions of ZO-1 and αSMA were substantially altered, confirming that the EMT of ARPE-19 cells was successfully induced by TGF-β2 treatment (S2 Fig). When we examined the expression levels of miR-21, miR-204, and let-7e in ARPE-19 at 48 h after TGF-β2 treatment, we found that the expression level of miR-21 was significantly upregulated by the TGF-β2 treatment, and the expression of miR-204 was significantly inhibited by TGF-β (Fig 3A and 3B). No significant change in let-7e expression after TGF-β treatment was observed (Fig 3C). These results suggested that the altered expression of miR-21 and miR-204 in PVD was associated with TGF-β-induced EMT of RPECs. Furthermore, we found that the expression levels of miR-21 in TGF-β-stimulated ARPE-19 cells were significantly higher under high-glucose conditions than low-glucose conditions (Fig 3D).

### MiR-21 Enhances Cell Migration and Proliferation of Retinal Pigment Epithelial Cells

The expression of miR-21 is often related to tumor progression in various types of cancers [32] and fibrosis [16–25], and has been reported to play a major pro-oncogenic and profibrotic role during disease progression. It is generally accepted as an oncomiR and fibromiR [33]. Thus, we next focused on miR-21 to further investigate the functional significance of this miRNA on fibroproliferative responses.

**Fig 4:**
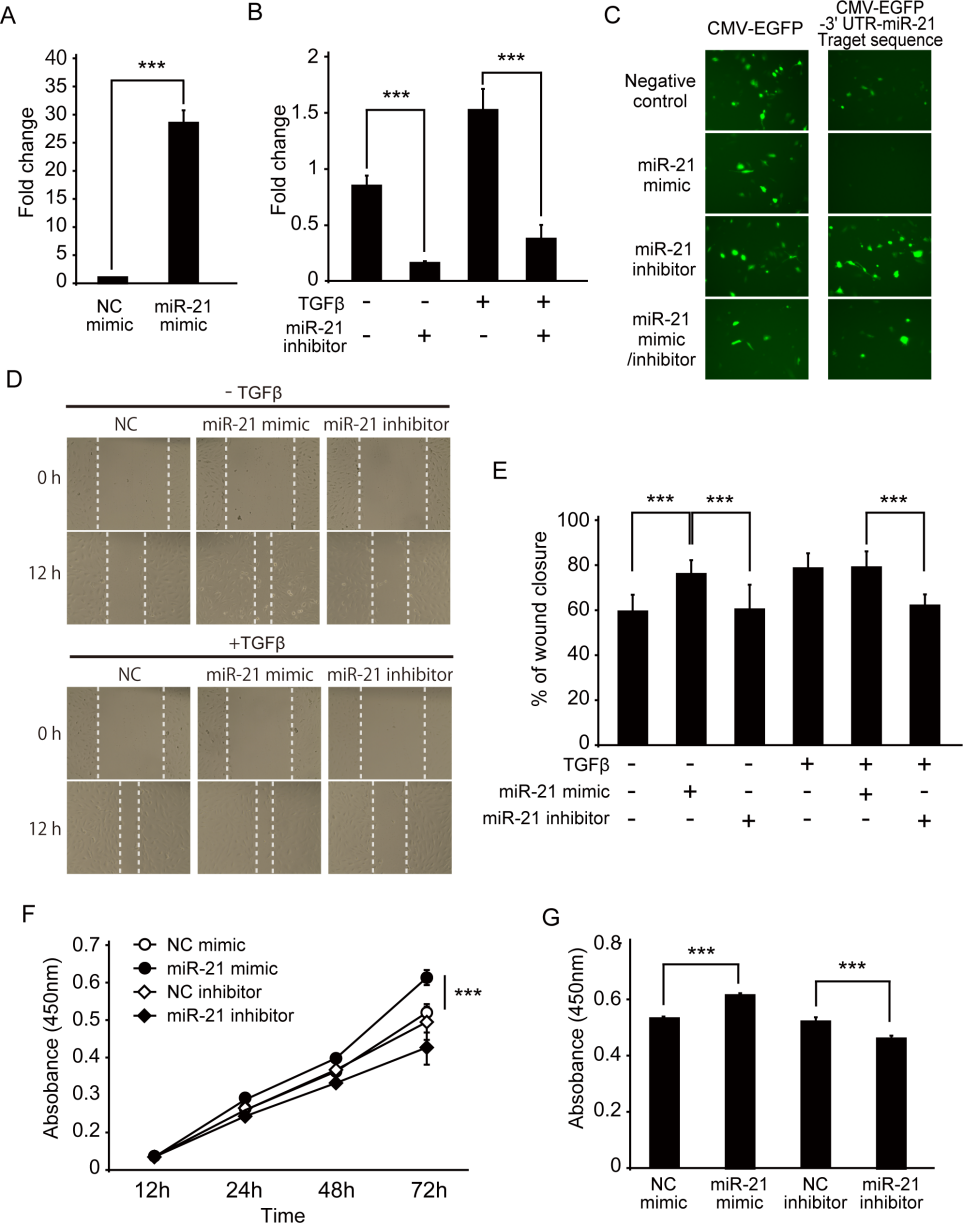
Effect of miR-21 expression on cell proliferation of ARPE-19 cells. (A, B) Results of quantitative (q)PCR analyses of miR-21 in miR-21 mimic- or inhibitor-transfected ARPE-19 cells. Fold changes in expression levels of miR-21 in miR-21 mimic-transfected ARPE-19 cells were determined relative to the negative control mimic-transfected cells (A). Expression level of miR-21 in miR-21 inhibitor-transfected ARPE-19 cells in the presence or absence of TGF-β2 was calculated by comparing with negative control inhibitor-transfected cells (B). (C) Effect of miR-21 mimic or miR-21 inhibitor transfection on GFP reporter gene expression of CMV-EGFP or CMV-EGFP-3′ UTR miR-21-target reporter plasmid in ARPE19 cells. ARPE-19 cells were co-transfected with CMV-EGFP or CMV-EGFP-3′ UTR miR-21-target reporter plasmid, together with miR-21 mimics or miR-21 inhibitor, as indicated. After 24 h, GFP expression was analyzed under the fluorescence microscope. All fluorescence images were taken at the same exposure time (1/15s). (D, E) The migration ability of miR-21 mimic and miR-21 inhibitor-treated ARPE-19 cells was examined using the wound healing assay. (D) Representative images of wound sealing of miR-21 mimic and miR-21 inhibitor-transfected cells cultured with or without TGF-β at 0 and 12 h. (E) Quantification of the wound healing assay. The figure shows the average percentage of wound closure ± SD. The transfection of the miR-21 mimic significantly increased cell migration of ARPE-19 cells without TGF-β stimulation, and transfection of miR-21 inhibitor inhibited TGF-β-induced enhanced migration. (F, G) Cell viability was measured with CCK-8 kits following the treatment of miR-21 mimic and miR-21 inhibitor for 12, 24, 48, 72, and 96 h (F). The miR-21 mimic treatment resulted in increased cell proliferation, and miR-21 inhibitor treatment resulted in decreased cell proliferation at 72 h (G). NC, negative control. ***p < 0.001; **p < 0.01; *p < 0.05.

To evaluate the potential role of miR-21 in cell proliferation and migration of RPECs, we performed gain- and loss-of-function studies using a miR-21 mimic/inhibitor. The results of real-time PCR showed that the expression level of miR-21 increased by approximately 30-fold in miR-21 mimic-transfected ARPE-19 cells compared with control cells, suggesting that there was approximately the same amount of induction of miR-21 expression in ARPE-19 cells as seen in the vitreous humor of PVR patients (Fig 4A). In addition, transfection with miR-21 inhibitor significantly downregulated the expression levels of both endogenous and TGF-β-induced miR-21 (Fig 4B). Furthermore, to confirm the specific effects of miR-21 mimic/inhibitor on ARPE-19 cells, GFP reporter constructs containing the miR-21 target sequence in the 3'-UTR were transfected with or without miR-21 mimic/inhibitor. After 24 h, GFP expression was significantly inhibited by miR-21 mimic and was successfully rescued by co-transfection with the miR-21 inhibitor (Fig 4C).

The wound healing assay for migration ability revealed that the migration ability of ARPE-19 cells was significantly enhanced by transfection with the miR-21 mimic (Fig 4D and 4E). In addition, TGF-β-induced enhancement of cell migration was significantly inhibited by the miR-21 inhibitor (Fig 4D and 4E).

Measurement of cell proliferation of the miR-21 mimic- or inhibitor-transfected ARPE-19 cells showed that the overexpression of miR-21 significantly promoted cell proliferation of cells at 72 h after transfection, whereas the miR-21 inhibitor-transfected cells were significantly inhibited (Fig 4F and 4G). Together, these results showed that miR-21 can regulate the cell migration and proliferation of RPECs during the fibroproliferative response.

### MiR-21 Does Not Affect the EMT of Retinal Pigment Epithelial Cells

**Fig 5:**
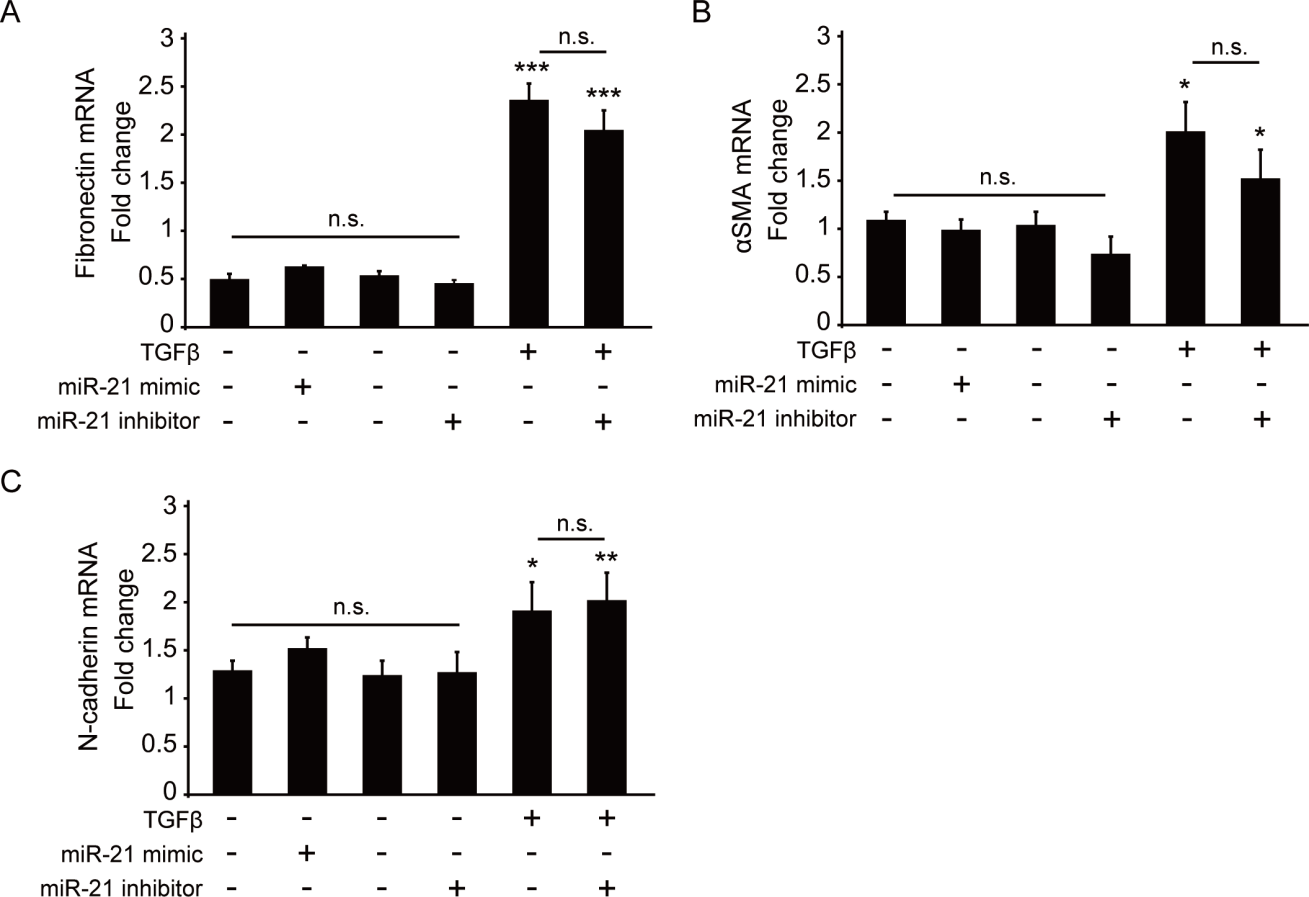
Effect of miR-21 expression on epithelial-mesenchymal transition (EMT)-related gene expression during TGF-β2-induced EMT of ARPE-19 cells. (A, B, and C) Results of qPCR analyses of fibronectin, αSMA, and N-cadherin mRNA expression in miR-21 mimic- or inhibitor-transfected ARPE-19 cells in the presence or absence of TGF-β2. Fold changes in expression levels of fibronectin (A), αSMA (B), and N-cadherin (C) mRNAs in miR-21 mimic-transfected ARPE-19 cells were determined relative to the untransfected control. NC, negative control; αSMA, alpha smooth muscle actin; EMT, epithelial-mesenchymal transition. ***p < 0.001; **p < 0.01; *p < 0.05; n.s; not significant.

To determine the function of miR-21 in the fibroproliferative response of RPECs, we examined the role of miR-21 on TGF-β-induced EMT of ARPE-19 cells by the gain- and loss-of-function approach. First, expression levels of EMT marker genes, such as genes for fibronectin, αSMA, and N-cadherin in the miR-21 mimic- or inhibitor-transfected ARPE-19 cells, were examined by quantitative RT-PCR (qRT-PCR). However, no change in the mRNA levels of these genes was observed (Fig 5A, 5B and 5C).

To examine the role of miR-21 on the expression level of the EMT-related genes during TGF-β-induced EMT, ARPE-19 cells were stimulated with TGF-β2 for 48 h, and mRNA expression levels of the EMT-related genes were examined by qRT-PCR. Although we observed significant upregulation of the expression levels of EMT-related genes by TGF-β2 stimulation, we could not observe any significant differences in the expression levels of all these genes between miR-21 mimic- or inhibitor-treated cells (Fig 5A, 5B and 5C). Together, these results suggested that miR-21 did not play a role in EMT induction of RPECs during the fibroproliferative response.

## Discussion

In this study, we identified the disease-associated miRNAs present in the vitreous humor of PVD samples using qRT-PCR-based expression profiling of 377 miRNAs. We observed the altered expression of various miRNAs in the vitreous humor from PVD eyes compared with that of MH samples. From these analyses, we identified and demonstrated that miR-21 is a potential disease-modifying miRNA involved in the development of PVD.

Ragusa et al. first reported that various miRNAs were present in human vitreous humor of MH, RRD, and uveal melanoma patients [12]. Tuo et al. performed global analyses of miRNA in the vitreous humor of primary vitreoretinal lymphoma and uveitis, and reported disease-related changes of expression of miR-155 [14]. However, it is still largely unknown how expression patterns of miRNAs in the vitreous humor differ between PVD and healthy eyes. In the present study, to identify specific miRNAs associated with fibroproliferative pathogenesis of PVD, we performed comprehensive expression analyses of miRNA in the vitreous humor of PVD patients, and focused on miR-21 and analyzed its function in RPECs.

Although we emphasized the expression of miR-21 in this study, we also observed a significant increase in the expression of miR-16, miR-92a, miR-130b, and miR-320. Previously, the upregulation of miR-16 and miR-92a was reported to be induced by vascular endothelial growth factor (VEGF) stimulation, and further reported to be functionally associated with the promotion of cell proliferation and migration of vascular endothelial cells [34, 35]. The expression level of VEGF is known to be upregulated in the vitreous of PDR eyes [36]. It is therefore possible that the upregulation of VEGF induced the expression of these miRNAs in the vitreous of PDR patients with active fibrovascular membrane eyes. Furthermore, the expression of miR-130b in serum has been reported to be associated with the development of type 2 diabetes mellitus. Thus, it is possible that the pathogenesis of diabetes in PDR causes the upregulation of the expression of miR-130b both in serum and in the vitreous of PDR eyes.

Among the downregulated miRNAs in the vitreous of PVD eyes, expression of miR-204 and let-7e has also been reported to decrease, and to be associated with the development of fibrosis of the lung or kidney [26, 27]. However, we found a significant reduction in miR-204 expression in the vitreous humor of RRD samples, suggesting that dedifferentiated RPECs may be involved in the downregulation of miR-204 expression [37].

We further demonstrated a significant increase in miR-21 expression in the vitreous humor of both PDR with active FVM and PVR eyes compared with control eyes. The expression of miR-21 in the vitreous humor of PVR eyes was significantly increased compared with that of the RRD eyes. Although the expression of miR-21 has been often associated with tumor progression in various types of cancers [32], it has been recently proposed that miR-21 plays a major role as a fibromiR in the development of cardiac, lung, and renal fibrosis [18, 24, 33, 38]. Notably, increased miR-21 levels in serum were also reported to be associated with kidney fibrosis [39]. It has been reported that expression of miR-21 in the vitreous humor of uveal melanoma was significantly higher than that in controls [12, 40]. Furthermore, elevated expression of miR-21 has also been reported in the rodent model of streptozotocin-induced diabetic retinopathy and ischemia-induced retinal neovascularization [41, 42], however a significant difference of miR-21 expression in the vitreous humor has not been observed between PDR and control eyes [13]. In addition, it has been reported that miR-21 is highly expressed in retinal microvascular endothelial cells [43]. It is therefore possible that a major source of miR-21 present in the vitreous humor is derived from not only RPECs, but also from retinal microvascular endothelial cells, which are also frequently upregulated in the eyes of PVD patients, especially in PDR patients. During this fibrosis process, it has been reported that the expression of miR-21 is strongly induced by TGF-β stimulation, which plays a central role in EMT by regulating fibrosis-related target gene expression [19, 22, 23]. Elevated glucose levels have been reported to induce miR-21 expression in diabetic mouse models and in some cultured cells, such as fibroblasts, glomerular cells, and renal tubular epithelial cells [25, 44–46]. This may play a critical role in organ fibrosis during the progression of diabetic complications as seen in diabetic nephropathy and diabetic gangrene. The roles of miR-21 in retinal fibroproliferative diseases have not been reported, and the present study is the first report of expression levels of miR-21 that are significantly induced by high glucose levels and by TGF-β2 stimulation in RPECs. In addition, we demonstrated that the overexpression of miR-21 significantly upregulated the cell migratory and proliferative ability of RPECs.

Circulating miRNAs in serum have been identified in CD63-positive exosomes and are believe to play a role in the regulation of gene expression of recipient cells by the transportation of microvesicles [47]. Recent studies reported that exosome secretion in RPECs is upregulated during the development of age-related macular degeneration, and it has been reported that stress induces the exocytotic activity of RPECs, suggesting that the miRNA-containing exosomes may be released to regulate the gene expression of surrounding cells [48, 49]. Because the elevated expression level of CD63, an exosomal marker, has been reported in stressed RPECs [48], it is possible that miR-21 containing exosomes are also released from RPECs during the development of PVD.

In summary, we have used qRT-PCR-based comprehensive analyses to identify the disease-associated miRNAs present in the vitreous humor of PVD eyes, and have identified miR-21 as a potential disease-modifying agent for the development of PVD. Our results suggest that the disease-associated expression of TGF-β2 and/or high glucose conditions enhance the expression of miR-21 in the vitreous humor of PVD eyes, which could play an important role in the fibroproliferative response of RPECs during the development of retinal fibrotic disorders.

## Supporting Information

S1 Fig Heat map of miRNA expression profiles from the vitreous humor of patients with PVD and MH. Clustering analyses of the expression patterns of microRNAs (miRNAs) in the vitreous humor of PVD (n = 3) and control MH (n = 3) samples. Red indicates high expression and green indicates low expression. Cluster 1 (downregulated miRNAs in PVR) is indicated in blue and cluster 2 (upregulated miRNAs in PVR) in red. PVD, proliferative vitreoretinal disease; MH, macular hole.
(TIF)

S2 Fig TGF-β2 induces EMT in ARPE-19 cells. (A) Phase contrast images of ARPE-19 cells untreated and treated with TGF-β2 (10 ng/mL); magnification, ×4, ×20. ARPE-19 cells after TGF-β2 treatment acquired spindle-type morphologies. (B) Immunofluorescence microscopy images showing the expression of ZO-1 (green) and αSMA (red) in ARPE-19 cells treated with or without TGFβ2. The nuclei were stained with 4',6-diamidino-2-phenylindole (DAPI) (blue). TGF-β2, transforming growth factor- β2; EMT, epithelial-mesenchymal transition.
(TIF)

S1 Table Results of integrated analyses of upregulated miRNAs in vitreous humors of PDR with PVR patients and downregulated mRNA expression profiles in human active fibrovascular membrane (FVM of PDR). PDR, proliferative diabetic retinopathy; PVR, proliferative vitreoretinopathy; FVM, fibrovascular membrane.
(DOCX)

S2 Table Integrated analyses of downregulated miRNAs in human vitreous of PDR with PVR and upregulated mRNA expression profiles in human active fibrovascular membrane (FVM of PDR). PDR, proliferative diabetic retinopathy; PVR, proliferative vitreoretinopathy.
(DOCX)

## References

[1] Abu El-Asrar AM, Van den Steen PE, Al-Amro SA, Missotten L, Opdenakker G, Geboes K. Expression of angiogenic and fibrogenic factors in proliferative vitreoretinal disorders. Int Ophthalmol. 2007;27(1):11–22. Epub 2007/03/22. 10.1007/s10792-007-9053-x . 17375263

[2] Saika S, Yamanaka O, Ikeda K, Kim-Mitsuyama S, Flanders KC, Yoo J, et al Inhibition of p38MAP kinase suppresses fibrotic reaction of retinal pigment epithelial cells. Lab Invest. 2005;85(7):838–50. Epub 2005/06/01. 10.1038/labinvest.3700294 . 15924151

[3] Kita T, Hata Y, Arita R, Kawahara S, Miura M, Nakao S, et al Role of TGF-beta in proliferative vitreoretinal diseases and ROCK as a therapeutic target. Proc Natl Acad Sci U S A. 2008;105(45):17504–9. Epub 2008/10/28. 10.1073/pnas.0804054105 ; PubMed Central PMCID: PMCPmc2582249. 18952846 PMC2582249

[4] Tosi GM, Marigliani D, Romeo N, Toti P. Disease pathways in proliferative vitreoretinopathy: an ongoing challenge. J Cell Physiol. 2014;229(11):1577–83. Epub 2014/03/08. 10.1002/jcp.24606 . 24604697

[5] Ha M, Kim VN. Regulation of microRNA biogenesis. Nature Reviews Molecular Cell Biology. 2014;15:509–24. 10.1038/nrm3838 25027649

[6] Weber JA, Baxter DH, Zhang S, Huang DY, Huang KH, Lee MJ, et al The microRNA spectrum in 12 body fluids. Clin Chem. 2010;56(11):1733–41. Epub 2010/09/18. 10.1373/clinchem.2010.147405 . 20847327 PMC4846276

[7] Valadi H, Ekstrom K, Bossios A, Sjostrand M, Lee JJ, Lotvall JO. Exosome-mediated transfer of mRNAs and microRNAs is a novel mechanism of genetic exchange between cells. Nat Cell Biol. 2007;9(6):654–9. Epub 2007/05/09. 10.1038/ncb1596 . 17486113

[8] Rayner KJ, Hennessy EJ. Extracellular communication via microRNA: lipid particles have a new message. J Lipid Res. 2013;54(5):1174–81. Epub 2013/03/19. 10.1194/jlr.R034991 ; PubMed Central PMCID: PMCPmc3622315. 23505318 PMC3622315

[9] Schwarzenbach H, Nishida N, Calin GA, Pantel K. Clinical relevance of circulating cell-free microRNAs in cancer. Nature Reviews Clinical Oncology. 2014;11:145–56. 10.1038/nrclinonc.2014.5 24492836

[10] Croce CM. Causes and consequences of microRNA dysregulation in cancer. Nat Rev Genet. 2009;10(10):704–14. Epub 2009/09/19. 10.1038/nrg2634 19763153 PMC3467096

[11] Dunmire JJ, Lagouros E, Bouhenni RA, Jones M, Edward DP. MicroRNA in aqueous humor from patients with cataract. Exp Eye Res. 2013;108:68–71. Epub 2012/11/14. 10.1016/j.exer.2012.10.016 . 23146683

[12] Ragusa M, Caltabiano R, Russo A, Puzzo L, Avitabile T, Longo A, et al MicroRNAs in vitreus humor from patients with ocular diseases. Mol Vis. 2013;19:430–40. 23441115 PMC3580974

[13] Hirota K, Keino H, Inoue M, Ishida H, Hirakata A. Comparisons of microRNA expression profiles in vitreous humor between eyes with macular hole and eyes with proliferative diabetic retinopathy. Graefes Arch Clin Exp Ophthalmol. 2014 10.1007/s00417-014-2692-5 . 24970617

[14] Tuo J, Shen D, Yang HH, Chan CC. Distinct microRNA-155 expression in the vitreous of patients with primary vitreoretinal lymphoma and uveitis. Am J Ophthalmol. 2014;157(3):728–34. Epub 2013/12/19. 10.1016/j.ajo.2013.12.014 ; PubMed Central PMCID: PMCPmc3961580. 24345320 PMC3961580

[15] Dunn KC, Aotaki-Keen AE, Putkey FR, Hjelmeland LM. ARPE-19, a human retinal pigment epithelial cell line with differentiated properties. Exp Eye Res. 1996;62(2):155–69. Epub 1996/02/01. 10.1006/exer.1996.0020 . 8698076

[16] Ardite E, Perdiguero E, Vidal B, Gutarra S, Serrano AL, Munoz-Canoves P. PAI-1-regulated miR-21 defines a novel age-associated fibrogenic pathway in muscular dystrophy. J Cell Biol. 2012;196(1):163–75. Epub 2012/01/04. 10.1083/jcb.201105013 22213800 PMC3255978

[17] Zhao J, Tang N, Wu K, Dai W, Ye C, Shi J, et al MiR-21 simultaneously regulates ERK1 signaling in HSC activation and hepatocyte EMT in hepatic fibrosis. PLoS One. 2014;9(10):e108005 Epub 2014/10/11. 10.1371/journal.pone.0108005 25303175 PMC4193742

[18] Thum T, Gross C, Fiedler J, Fischer T, Kissler S, Bussen M, et al MicroRNA-21 contributes to myocardial disease by stimulating MAP kinase signalling in fibroblasts. Nature. 2008;456(7224):980–4. 10.1038/nature07511 19043405

[19] Bronnum H, Andersen DC, Schneider M, Sandberg MB, Eskildsen T, Nielsen SB, et al miR-21 promotes fibrogenic epithelial-to-mesenchymal transition of epicardial mesothelial cells involving Programmed Cell Death 4 and Sprouty-1. PLoS One. 2013;8(2):e56280 Epub 2013/02/27. 10.1371/journal.pone.0056280 23441172 PMC3575372

[20] Liu G, Friggeri A, Yang Y, Milosevic J, Ding Q, Thannickal VJ, et al miR-21 mediates fibrogenic activation of pulmonary fibroblasts and lung fibrosis. J Exp Med. 2010;207(8):1589–97. Epub 2010/07/21. 10.1084/jem.20100035 20643828 PMC2916139

[21] Zhou Y, Xiong M, Fang L, Jiang L, Wen P, Dai C, et al miR-21-containing microvesicles from injured tubular epithelial cells promote tubular phenotype transition by targeting PTEN protein. Am J Pathol. 2013;183(4):1183–96. Epub 2013/08/28. 10.1016/j.ajpath.2013.06.032 . 23978520

[22] Zhu H, Luo H, Li Y, Zhou Y, Jiang Y, Chai J, et al MicroRNA-21 in scleroderma fibrosis and its function in TGF-beta-regulated fibrosis-related genes expression. J Clin Immunol. 2013;33(6):1100–9. Epub 2013/05/10. 10.1007/s10875-013-9896-z . 23657402

[23] Kumarswamy R, Volkmann I, Jazbutyte V, Dangwal S, Park DH, Thum T. Transforming growth factor-beta-induced endothelial-to-mesenchymal transition is partly mediated by microRNA-21. Arterioscler Thromb Vasc Biol. 2012;32(2):361–9. Epub 2011/11/19. 10.1161/atvbaha.111.234286 . 22095988

[24] Yamada M, Kubo H, Ota C, Takahashi T, Tando Y, Suzuki T, et al The increase of microRNA-21 during lung fibrosis and its contribution to epithelial-mesenchymal transition in pulmonary epithelial cells. Respir Res. 2013;14:95 Epub 2013/09/26. 10.1186/1465-9921-14-95 24063588 PMC3849377

[25] Wang J, Gao Y, Ma M, Li M, Zou D, Yang J, et al Effect of miR-21 on renal fibrosis by regulating MMP-9 and TIMP1 in kk-ay diabetic nephropathy mice. Cell Biochem Biophys. 2013;67(2):537–46. Epub 2013/02/28. 10.1007/s12013-013-9539-2 . 23443810

[26] Scian MJ, Maluf DG, David KG, Archer KJ, Suh JL, Wolen AR, et al MicroRNA profiles in allograft tissues and paired urines associate with chronic allograft dysfunction with IF/TA. Am J Transplant. 2011;11(10):2110–22. Epub 2011/07/29. 10.1111/j.1600-6143.2011.03666.x ; PubMed Central PMCID: PMCPmc3184368. 21794090 PMC3184368

[27] Vettori S, Gay S, Distler O. Role of MicroRNAs in Fibrosis. Open Rheumatol J. 2012;6:130–9. Epub 2012/07/18. 10.2174/1874312901206010130 22802911 PMC3396185

[28] Pandit KV, Milosevic J, Kaminski N. MicroRNAs in idiopathic pulmonary fibrosis. Transl Res. 2011;157(4):191–9. Epub 2011/03/23. 10.1016/j.trsl.2011.01.012 . 21420029

[29] Chen PY, Qin L, Barnes C, Charisse K, Yi T, Zhang X, et al FGF regulates TGF-beta signaling and endothelial-to-mesenchymal transition via control of let-7 miRNA expression. Cell Rep. 2012;2(6):1684–96. Epub 2012/12/04. 10.1016/j.celrep.2012.10.021 23200853 PMC3534912

[30] Pastor JC, de la Rúa ER, Martín F. Proliferative vitreoretinopathy: risk factors and pathobiology. Prog Retin Eye Res. 2002;21(1):127–44. . 11906814 10.1016/s1350-9462(01)00023-4

[31] Antonetti DA, Klein R, Gardner TW. Diabetic retinopathy. N Engl J Med. 2012;366(13):1227–39. Epub 2012/03/30. 10.1056/NEJMra1005073 . 22455417

[32] Volinia S, Calin GA, Liu CG, Ambs S, Cimmino A, Petrocca F, et al A microRNA expression signature of human solid tumors defines cancer gene targets. Proc Natl Acad Sci U S A. 2006;103(7):2257–61. Epub 2006/02/08. 10.1073/pnas.0510565103 16461460 PMC1413718

[33] Pottier N, Cauffiez C, Perrais M, Barbry P, Mari B. FibromiRs: translating molecular discoveries into new anti-fibrotic drugs. Trends Pharmacol Sci. 2014;35(3):119–26. Epub 2014/02/25. 10.1016/j.tips.2014.01.003 . 24560301

[34] Chamorro-Jorganes A, Araldi E, Penalva LO, Sandhu D, Fernandez-Hernando C, Suarez Y. MicroRNA-16 and microRNA-424 regulate cell-autonomous angiogenic functions in endothelial cells via targeting vascular endothelial growth factor receptor-2 and fibroblast growth factor receptor-1. Arterioscler Thromb Vasc Biol. 2011;31(11):2595–606. Epub 2011/09/03. 10.1161/atvbaha.111.236521 21885851 PMC3226744

[35] Chang SH, Hla T. Gene regulation by RNA binding proteins and microRNAs in angiogenesis. Trends Mol Med. 2011;17(11):650–8. Epub 2011/08/02. 10.1016/j.molmed.2011.06.008 21802991 PMC3625859

[36] Burgos R, Simo R, Audi L, Mateo C, Mesa J, Garcia-Ramirez M, et al Vitreous levels of vascular endothelial growth factor are not influenced by its serum concentrations in diabetic retinopathy. Diabetologia. 1997;40(9):1107–9. Epub 1997/09/23. 10.1007/s001250050794 . 9300249

[37] Adijanto J, Castorino JJ, Wang ZX, Maminishkis A, Grunwald GB, Philp NJ. Microphthalmia-associated transcription factor (MITF) promotes differentiation of human retinal pigment epithelium (RPE) by regulating microRNAs-204/211 expression. J Biol Chem. 2012;287(24):20491–503. Epub 2012/04/24. 10.1074/jbc.M112.354761 22523078 PMC3370234

[38] Gomez IG, MacKenna DA, Johnson BG, Kaimal V, Roach AM, Ren S, et al Anti-microRNA-21 oligonucleotides prevent Alport nephropathy progression by stimulating metabolic pathways. J Clin Invest. 2015;125(1):141–56. Epub 2014/11/22. 10.1172/jci75852 25415439 PMC4382246

[39] Glowacki F, Savary G, Gnemmi V, Buob D, Van der Hauwaert C, Lo-Guidice JM, et al Increased circulating miR-21 levels are associated with kidney fibrosis. PloS one. 2013;8(2):e58014 10.1371/journal.pone.0058014 23469132 PMC3585177

[40] Ragusa M, Barbagallo C, Statello L, Caltabiano R, Russo A, Puzzo L, et al miRNA profiling in vitreous humor, vitreal exosomes and serum from uveal melanoma patients: Pathological and diagnostic implications. Cancer Biol Ther. 2015:0. Epub 2015/05/08. 10.1080/15384047.2015.1046021 . 25951497 PMC4622662

[41] Kovacs B, Lumayag S, Cowan C, Xu S. MicroRNAs in early diabetic retinopathy in streptozotocin-induced diabetic rats. Invest Ophthalmol Vis Sci. 2011;52(7):4402–9. 10.1167/iovs.10-6879 . 21498619

[42] Shen J, Yang X, Xie B, Chen Y, Swaim M, Hackett SF, et al MicroRNAs regulate ocular neovascularization. Mol Ther. 2008;16(7):1208–16. 10.1038/mt.2008.104 18500251 PMC3033219

[43] Guduric-Fuchs J, O'Connor A, Cullen A, Harwood L, Medina RJ, O'Neill CL, et al Deep sequencing reveals predominant expression of miR-21 amongst the small non-coding RNAs in retinal microvascular endothelial cells. J Cell Biochem. 2012;113(6):2098–111. 10.1002/jcb.24084 22298343 PMC3708110

[44] Zhong X, Chung AC, Chen HY, Dong Y, Meng XM, Li R, et al miR-21 is a key therapeutic target for renal injury in a mouse model of type 2 diabetes. Diabetologia. 2013;56(3):663–74. Epub 2013/01/08. 10.1007/s00125-012-2804-x . 23292313

[45] Madhyastha R, Madhyastha H, Pengjam Y, Nakajima Y, Omura S, Maruyama M. NFkappaB activation is essential for miR-21 induction by TGFbeta1 in high glucose conditions. Biochem Biophys Res Commun. 2014;451(4):615–21. Epub 2014/08/19. 10.1016/j.bbrc.2014.08.035 . 25130469

[46] Dey N, Ghosh-Choudhury N, Kasinath BS, Choudhury GG. TGFbeta-stimulated microRNA-21 utilizes PTEN to orchestrate AKT/mTORC1 signaling for mesangial cell hypertrophy and matrix expansion. PLoS One. 2012;7(8):e42316 Epub 2012/08/11. 10.1371/journal.pone.0042316 22879939 PMC3411779

[47] Kosaka N, Yoshioka Y, Hagiwara K, Tominaga N, Katsuda T, Ochiya T. Trash or Treasure: extracellular microRNAs and cell-to-cell communication. Front Genet. 2013;4:173 10.3389/fgene.2013.00173 24046777 PMC3763217

[48] Biasutto L, Chiechi A, Couch R, Liotta LA, Espina V. Retinal pigment epithelium (RPE) exosomes contain signaling phosphoproteins affected by oxidative stress. Exp Cell Res. 2013;319(13):2113–23. 10.1016/j.yexcr.2013.05.005 23669273 PMC3727419

[49] Wang AL, Lukas TJ, Yuan M, Du N, Tso MO, Neufeld AH. Autophagy and exosomes in the aged retinal pigment epithelium: possible relevance to drusen formation and age-related macular degeneration. PLoS One. 2009;4(1):e4160 10.1371/journal.pone.0004160 19129916 PMC2612751

