# Supplementary figures and images for: Upregulation of Mir-21 Levels in the Vitreous Humor Is Associated with Development of Proliferative Vitreoretinal Disease
Source: PLoS One. 2016 Jun 28;11(6):e0158043. doi: 10.1371/journal.pone.0158043 (PMC4924816; doi:10.1371/journal.pone.0158043)

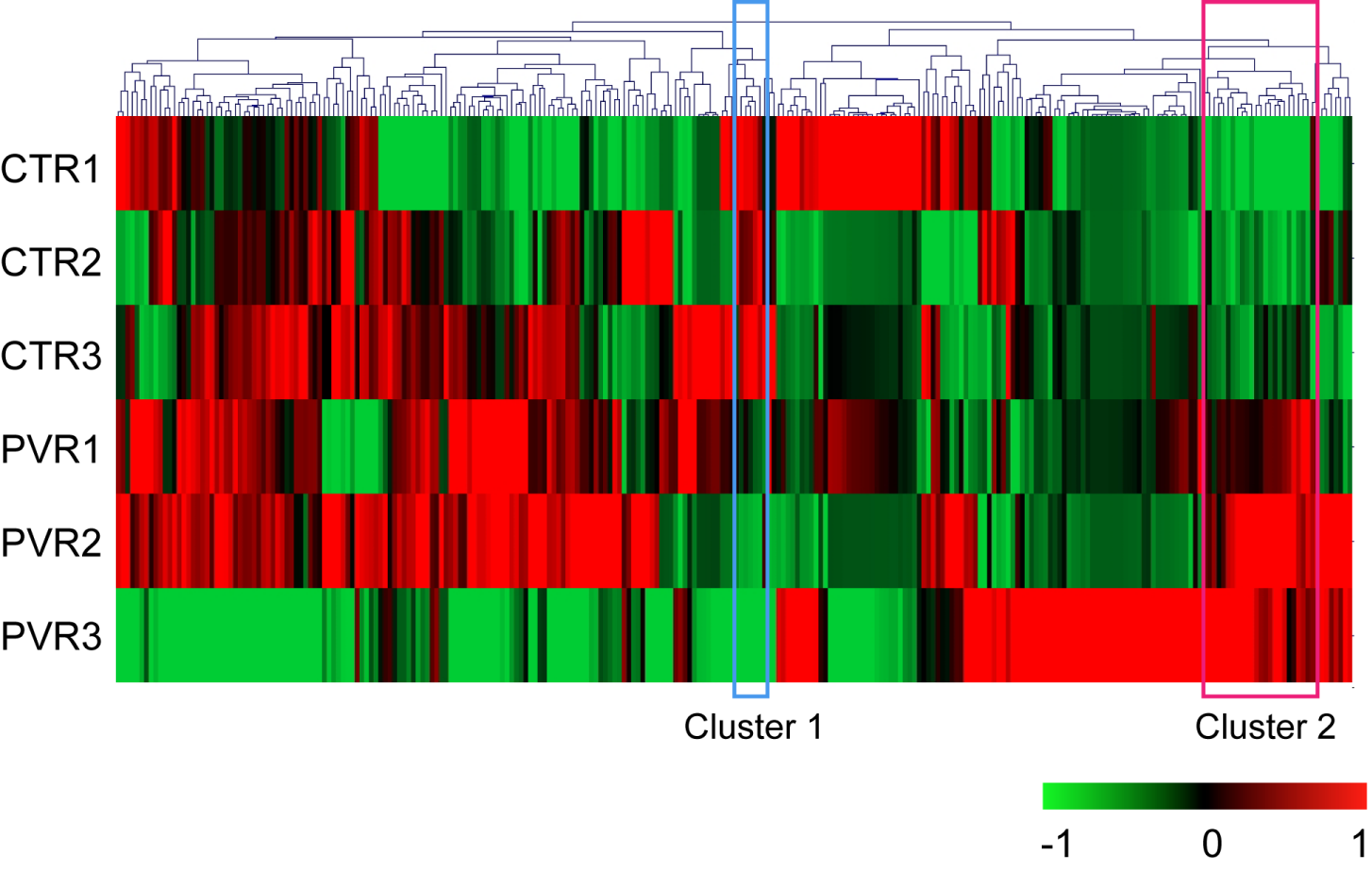

Supplement: S1 Fig — Clustering analyses of the expression patterns of microRNAs (miRNAs) in the vitreous humor of PVD (n = 3) and control MH (n = 3) samples. Red indicates high expression and green indicates low expression. Cluster 1 (downregulated miRNAs in PVR) is indicated in blue and cluster 2 (upregulated miRNAs in PVR) in red. PVD, proliferative vitreoretinal disease; MH, macular hole. (TIF) [file pone.0158043.s001.tif]

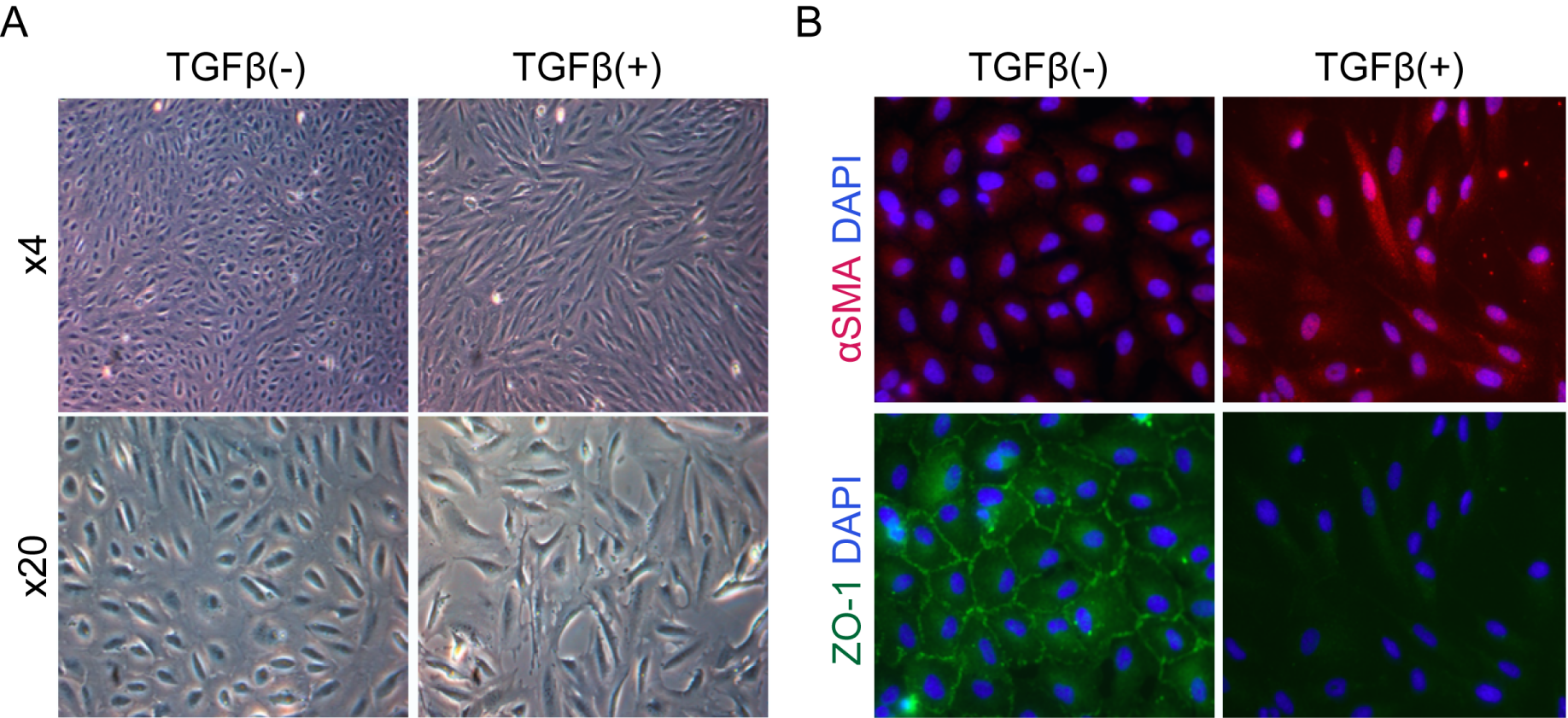

Supplement: S2 Fig — (A) Phase contrast images of ARPE-19 cells untreated and treated with TGF-β2 (10 ng/mL); magnification, ×4, ×20. ARPE-19 cells after TGF-β2 treatment acquired spindle-type morphologies. (B) Immunofluorescence microscopy images showing the expression of ZO-1 (green) and αSMA (red) in ARPE-19 cells treated with or without TGFβ2. The nuclei were stained with 4',6-diamidino-2-phenylindole (DAPI) (blue). TGF-β2, transforming growth factor- β2; EMT, epithelial-mesenchymal transition. (TIF) [file pone.0158043.s002.tif]
