# Supplementary material for: Upregulation of Mir-21 Levels in the Vitreous Humor Is Associated with Development of Proliferative Vitreoretinal Disease
Source: PLoS One. 2016 Jun 28;11(6):e0158043. doi: 10.1371/journal.pone.0158043 (PMC4924816; doi:10.1371/journal.pone.0158043)
Supplement: S1 Table — PDR, proliferative diabetic retinopathy; PVR, proliferative vitreoretinopathy; FVM, fibrovascular membrane. (DOCX) [file pone.0158043.s003.docx]

**S Table 1**

|  | Target gene | miRNA | | | | | | |
| --- | --- | --- | --- | --- | --- | --- | --- | --- |
| 1 | AOX1 | miR-16 | miR-27b |  |  |  |  |  |
| 2 | BMP6 | miR-16 | miR-27b | miR-106b | miR-130b | miR-210 |  |  |
| 3 | BMP8B | miR-16 | miR-27b | miR-106b | miR-130b | miR-210 |  |  |
| 4 | C1QTNF3 | miR-16 | miR-92a | miR-106b |  |  |  |  |
| 5 | C20orf39 | miR-16 | miR-92a | miR-210 |  |  |  |  |
| 6 | COL4A5 | miR-16 | miR-21 | miR-92a | miR-106b |  |  |  |
| 7 | ENSG00000011177 | miR-16 |  |  |  |  |  |  |
| 8 | ENSG00000011177 | miR-16 | miR-21 | miR-27b | miR-92a | miR-210 |  |  |
| 9 | ENSG00000011465 | miR-16 | miR-21 | miR-92a |  |  |  |  |
| 10 | ENSG00000012171 | miR-16 | miR-27b | miR-92a | miR-210 |  |  |  |
| 11 | ENSG00000064886 | miR-16 |  |  |  |  |  |  |
| 12 | ENSG00000104894 | miR-16 |  |  |  |  |  |  |
| 13 | ENSG00000107130 | miR-16 | miR-27b | miR-106b | miR-130b | miR-210 |  |  |
| 14 | ENSG00000162777 | miR-16 | miR-106b |  |  |  |  |  |
| 15 | ENSG00000162946 | miR-16 | miR-21 | miR-27b | miR-92a | miR-106b | miR-210 |  |
| 16 | ENSG00000173068 | miR-16 | miR-21 | miR-27b | miR-92a | miR-106b | miR-130b | miR-210 |
| 17 | FMO2 | miR-16 | miR-21 | miR-27b | miR-92a |  |  |  |
| 18 | FNDC1 | miR-16 | miR-21 | miR-27b |  |  |  |  |
| 19 | ISLR | miR-16 | miR-27b |  |  |  |  |  |
| 20 | LGI4 | miR-16 | miR-27b | miR-210 |  |  |  |  |
| 21 | MFAP4 | miR-16 | miR-21 | miR-210 |  |  |  |  |
| 22 | MN1 | miR-16 | miR-27b | miR-106b | miR-130b |  |  |  |
| 23 | NLGN4X | miR-16 | miR-27b | miR-92a | miR-106b | miR-130b | miR-210 |  |
| 24 | OSR1 | miR-16 | miR-21 | miR-106b |  |  |  |  |
| 25 | PLP1 | miR-16 | miR-21 | miR-92a |  |  |  |  |
| 26 | PRELP | miR-16 | miR-21 | miR-27b | miR-106b |  |  |  |
| 27 | RASSF5 | miR-16 | miR-21 | miR-27b | miR-106b | miR-130b | miR-210 |  |
| 28 | SFRP4 | miR-16 | miR-92a |  |  |  |  |  |
| 29 | USH1C | miR-16 |  |  |  |  |  |  |
| 30 | ZC3H12B | miR-16 | miR-21 | miR-27b | miR-92a | miR-106b | miR-130b | miR-210 |
| 31 | BMP6 | miR-21 |  |  |  |  |  |  |
| 32 | CD48 | miR-21 | miR-27b | miR-92a |  |  |  |  |
| 33 | CDKN2B | miR-21 | miR-92a | miR-106b | miR-130b | miR-210 |  |  |
| 34 | ENSG00000079931 | miR-21 | miR-27b | miR-92a |  |  |  |  |
| 35 | ENSG00000109667 | miR-21 | miR-106b |  |  |  |  |  |
| 36 | FYB | miR-21 | miR-106b |  |  |  |  |  |
| 37 | GAS1 | miR-21 |  |  |  |  |  |  |
| 38 | RASGRP1 | miR-21 | miR-27b | miR-106b | miR-130b | miR-210 |  |  |
| 39 | SMOC2 | miR-21 | miR-27b | miR-92a | miR-106b | miR-130b |  |  |
| 40 | TGM2 | miR-21 | miR-27b | miR-92a | miR-130b | miR-210 |  |  |
| 41 | TMEM178 | miR-21 | miR-27b | miR-92a | miR-130b | miR-210 |  |  |
| 42 | ZFP36L2 | miR-21 |  |  |  |  |  |  |
| 43 | ENSG00000011177 | miR-27b |  |  |  |  |  |  |
| 44 | ENSG00000117643 | miR-27b | miR-106b | miR-130b |  |  |  |  |
| 45 | ENSG00000154262 | miR-27b | miR-92a |  |  |  |  |  |
| 46 | FBLN2 | miR-27b | miR-130b |  |  |  |  |  |
| 47 | FMOD | miR-27b | miR-92a | miR-106b |  |  |  |  |
| 48 | GFRA1 | miR-27b |  |  |  |  |  |  |
| 49 | GPM6B | miR-27b | miR-106b |  |  |  |  |  |
| 50 | EDG2 | miR-92a | miR-130b |  |  |  |  |  |
| 51 | ENSG00000011177 | miR-92a |  |  |  |  |  |  |
| 52 | ENSG00000127083 | miR-92a |  |  |  |  |  |  |
| 53 | NR1H4 | miR-92a |  |  |  |  |  |  |
| 54 | ABCA8 | miR-106b |  |  |  |  |  |  |
| 55 | DHRS3 | miR-106b | miR-130b |  |  |  |  |  |
| 56 | NINJ2 | miR-106b |  |  |  |  |  |  |
| 57 | SEMA3C | miR-106b |  |  |  |  |  |  |
| 58 | ENSG00000011177 | miR-210 |  |  |  |  |  |  |
| 59 | ENSG00000126264 | miR-210 |  |  |  |  |  |  |
