# Supplementary material for: Upregulation of Mir-21 Levels in the Vitreous Humor Is Associated with Development of Proliferative Vitreoretinal Disease
Source: PLoS One. 2016 Jun 28;11(6):e0158043. doi: 10.1371/journal.pone.0158043 (PMC4924816; doi:10.1371/journal.pone.0158043)
Supplement: S2 Table — PDR, proliferative diabetic retinopathy; PVR, proliferative vitreoretinopathy. (DOCX) [file pone.0158043.s004.docx]

**S Table 2**

|  | | Target gene | miRNA | | | | | |
| --- | --- | --- | --- | --- | --- | --- | --- | --- |
| 1 | | C16orf30 | miR-199a-3p | miR-204 |  |  |  |  |
| 2 | | C21orf7 | miR-9 | let-7e | miR-204 | mir-216b | |  |
| 3 | | CDH5 | miR-9 | miR-139 | miR-199a-3p | miR-204 | let-7e |  |
| 4 | COL18A1 | | miR-9 | miR-204 | mir-216b | |  |  |
| 5 | DLL4 | | miR-199a-3p | miR-204 | mir-216b | let-7e |  |  |
| 6 | DPYSL4 | | miR-139 | miR-204 |  |  |  |  |
| 7 | EFNA1 | | miR-9 | miR-204 | let-7e |  |  |  |
| 8 | ENSG00000011052 | | miR-139 | miR-204 |  |  |  |  |
| 9 | ENSG00000080709 | | miR-199a-3p | miR-204 |  |  |  |  |
| 10 | ENSG00000106125 | | miR-9 | miR-204 | miR-199a-3p | miR-139 | mir-216b | |
| 11 | ENSG00000163638 | | miR-9 | miR-204 | miR-139 | mir-216b |  | |
| 12 | ENSG00000188229 | | miR-204 |  |  |  |  |  |
| 13 | ENSG00000198794 | | miR-9 | miR-204 | miR-139 | let-7e |  |  |
| 14 | ETS1 | | miR-9 | miR-139 | miR-199a-3p | miR-204 | mir-216b | |
| 15 | FLNA | | miR-9 | miR-139 | miR-199a-3p | miR-204 | let-7e |  |
| 16 | GPX7 | | miR-9 | miR-139 | miR-204 | let-7e |  |  |
| 17 | GSTM3 | | miR-9 | miR-204 | miR-139 | mir-216b |  | |
| 18 | HEYL | | miR-9 | miR-199a-3p | miR-204 | mir-216b | let-7e |  |
| 19 | HSD11B2 | | miR-199a-3p | miR-204 |  |  |  |  |
| 20 | KCNAB2 | | miR-9 | miR-139 | miR-204 | mir-216b | let-7e |  |
| 21 | KCNJ2 | | miR-9 | miR-139 | miR-204 | mir-216b | let-7e |  |
| 22 | KCNJ8 | | miR-139 | miR-199a-3p | miR-204 |  |  |  |
| 23 | LZTS1 | | miR-9 | miR-139 | miR-204 | mir-216b | let-7e |  |
| 24 | MARCH4 | | miR-9 | miR-204 | mir-216b | |  |  |
| 25 | MCAM | | miR-9 | miR-204 | let-7e |  |  |  |
| 26 | MTSS1 | | miR-139 | miR-199a-3p | miR-204 | mir-216b |  | |
| 27 | MYO1B | | miR-9 | miR-139 | miR-204 |  |  |  |
| 28 | NRXN2 | | miR-204 |  |  |  |  |  |
| 29 | OLFML2A | | miR-9 | miR-139 | miR-199a-3p | miR-204 | mir-216b | let-7e |
| 30 | PCDH12 | | miR-9 | miR-139 | miR-204 |  |  |  |
| 31 | PDGFB | | miR-9 | miR-139 | miR-199a-3p | miR-204 | mir-216b | let-7e |
| 32 | PLVAP | | miR-139 | miR-204 | mir-216b | |  |  |
| 33 | PTPRM | | miR-9 | miR-204 | mir-216b | |  |  |
| 34 | RARB | | miR-9 | miR-199a-3p | miR-204 | mir-216b | let-7e |  |
| 35 | RASSF2 | | miR-9 | miR-139 | miR-199a-3p | miR-204 | mir-216b | let-7e |
| 36 | SEMA3F | | miR-9 | miR-139 | miR-204 | let-7e |  |  |
| 37 | SPRY4 | | miR-9 | miR-139 | miR-199a-3p | miR-204 | let-7e |  |
| 38 | TGFB3 | | miR-199a-3p | miR-204 | let-7e |  |  |  |
| 39 | TNFAIP6 | | miR-204 |  |  |  |  |  |
| 40 | TNFAIP8L1 | | miR-139 | miR-199a-3p | miR-204 | mir-216b | let-7e |  |
| 41 | WDR51A | | miR-9 | miR-139 | miR-204 | mir-216b | let-7e |  |
| 42 | C15orf39 | | miR-9 | miR-139 | miR-199a-3p | let-7e |  |  |
| 43 | DOCK6 | | miR-9 |  |  |  |  |  |
| 44 | ESM1 | | miR-9 | miR-139 | mir-216b | let-7e |  |  |
| 45 | NID2 | | miR-9 | miR-139 | miR-199a-3p | mir-216b | let-7e |  |
| 46 | PPEF1 | | miR-9 | miR-199a-3p | |  |  |  |
| 47 | SH2D3C | | miR-9 | miR-199a-3p | mir-216b | |  |  |
| 48 | TSPAN18 | | miR-9 | miR-139 | mir-216b | let-7e |  |  |
| 49 | CETP | | mir-216b | |  |  |  |  |
| 50 | ENSG00000100453 | | mir-216b | |  |  |  |  |
| 51 | FAM19A3 | | mir-216b | |  |  |  |  |
| 52 | TACC2 | | miR-199a-3p | mir-216b | let-7e |  |  |  |
| 53 | DYSF | | miR-139 |  |  |  |  |  |
| 54 | CACNA1S | | let-7e |  |  |  |  |  |
